# Comparison of Postoperative Nausea and Vomiting Between Sedation with Remimazolam and Dexmedetomidine in Transcatheter Aortic Valve Replacement Patients: A Single-Center Retrospective Observational Study

**DOI:** 10.3390/jcm14051759

**Published:** 2025-03-05

**Authors:** Takashi Mino, Atsuhiro Kitaura, Hiroatsu Sakamoto, Yukari Yoshino, Shota Tsukimoto, Haruyuki Yuasa, Yasufumi Nakajima

**Affiliations:** 1 Department of Anesthesiology, Faculty of Medicine, Kindai University, 377-2 Ono-Higashi, Osakasayama 589-8511, Japan; takashi.mino@med.kindai.ac.jp (T.M.);; 2 Department of Anesthesiology, Kanagawa Dental University, 82 Inaoka, Yokosuka 238-0003, Japan; 3 Center for Outcomes Research, University of Texas Health Science Center, Huston, TX 77054, USA

**Keywords:** postoperative nausea and vomiting, remimazolam, dexmedetomidine, transcatheter aortic valve replacement, monitored anesthesia care, quality of recovery

## Abstract

**Background/Objectives**: Remimazolam, a short-acting benzodiazepine, promotes quick and consistent recovery from anesthesia. However, flumazenil’s rapid antagonistic effects on benzodiazepines during the emergence from anesthesia are thought to increase the risk of postoperative nausea and vomiting (PONV). This study aimed to compare the rate of PONV in monitored anesthesia care (MAC) with remimazolam versus conventional MAC with dexmedetomidine. **Methods**: This single-center retrospective study included all cases with transcatheter aortic valve replacements (TAVR) performed using MAC at our institution between January 2019 and April 2023. The patients were divided into remimazolam and dexmedetomidine–propofol groups based on the anesthetic method used. We used propensity score matching at a 1:1 ratio to account for the patient backgrounds. The primary outcome measure was the rate of PONV within 48 h. Secondary outcome measures included the severity of PONV and the number of antiemetics administered. **Results**: This study included 177 subjects. Following propensity score matching, 61 patients were allocated to each group. The incidence of PONV within 48 h after surgery was 4.92% in the remimazolam group and 3.28% in the dexmedetomidine–propofol group, with no significant difference between the two groups (*p* = 0.817). There was no significant difference between the two groups in terms of the secondary outcomes, including the severity of PONV (*p* = 0.190) and the use of antiemetics (*p* = 0.690). **Conclusions**: In TAVR with MAC and remimazolam, the incidence of PONV within 48 h was comparable to that of dexmedetomidine.

## 1. Introduction

Remimazolam, a short-acting benzodiazepine sedative, is developed for sedation and general anesthesia [1,2]. It is distinguished by its minimal effect on hemodynamics, which is typical of benzodiazepine anesthetics [1], as well as its short half-life when compared to conventional benzodiazepine anesthetics [1], making it simple to administer. Furthermore, the combination of remimazolam and flumazenil may result in faster arousal, a shorter anesthesia time [3], and potentially improved postoperative recovery [4]. Considering the characteristics of remimazolam, our research team has actively utilized it in high-risk elderly patients with severe aortic stenosis who are also frail. We used remimazolam, flumazenil, and remifentanil for sedation in transcatheter aortic valve replacement (TAVR) procedures. Our sedation method, when compared to the conventional method using dexmedetomidine–propofol, has been shown to provide stable hemodynamics and shorter awakening times, as well as a favorable intraoperative environment [3]. However, our previous study did not consider PONV, which could be a significant complication [3], and, based on our clinical experience, we did not believe that flumazenil’s acute antagonism caused PONV in the same way that previous studies suggested. On the other hand, the rapid antagonism of benzodiazepines is thought to increase the risk of postoperative nausea and vomiting (PONV) [5]. PONV is defined as a postoperative complication that occurs within 0–48 h of general anesthesia [6,7,8,9,10]. Several reports have indicated an increase in PONV. PONV slows down patients’ postoperative recovery, prevents oral intake and early ambulation, and can be more distressing than wound pain. As a result, PONV extends hospital stays and significantly raises healthcare costs [11,12,13]. Therefore, managing PONV is critical given the benefits to patients and the societal implications. There are several reports on the risk of PONV associated with remimazolam. Compared to inhalational anesthetics, remimazolam is reported to either reduce PONV or have comparable levels [4,14]. However, when compared to intravenous anesthetics, conflicting reports have been made regarding the risk of PONV with remimazolam. In studies comparing remimazolam and propofol, higher incidence rates of PONV were found with remimazolam [15,16], whereas others have suggested that remimazolam and propofol did not increase the frequency of PONV [17,18,19]. In previous studies, research reporting an increase in PONV with remimazolam included the use of flumazenil, while studies reporting no increase in PONV with remimazolam tended to not use flumazenil [15,16,17,18,19]. Therefore, in this study, it was necessary to confirm that our remimazolam-based protocol did not cause issues regarding the incidence of postoperative nausea and vomiting (PONV) in order to validate its effectiveness. Remimazolam is still a new drug, and there is a lack of evidence regarding specific patient populations, so the accumulation of evidence is desirable [20]. To the best of our knowledge, there are no studies specifically addressing PONV in TAVR procedures using remimazolam, nor studies focused on elderly patients. While elderly patients are generally considered to be in a low-risk category for PONV, publishing data on this patient group, where the characteristics of remimazolam are thought to be the most beneficial, would contribute to enhancing the safety profile of remimazolam.

In this study, we established a null hypothesis that MAC using remimazolam, remifentanil, and flumazenil would result in increased PONV compared to the standard MAC regimen of dexmedetomidine, propofol, and remifentanil in TAVR patients.

## 2. Materials and Methods

This study was a single-center retrospective observational study based on our institution’s (Kindai University Hospital, Osaka-Sayama, Japan) electronic medical records, approved by the Institutional Review Board (IRB) of Kindai University Faculty of Medicine (protocol code: R05-179 and date of approval: 12 March 2024). We used the opt-out model. All patients who underwent transfemoral TAVR under MAC at Kindai University Hospital between January 2019 and April 2023 were retrospectively identified using our hospital’s electronic medical records, without first calculating the sample size. Our institution used the HOPE LifeMark-HX electronic medical record system (Fujitsu Ltd., Tokyo, Japan) and the Prime Gaia electronic anesthesia record system (Nihon Kohden Co., Tokyo, Japan). Initially, patients who underwent TAVR during the study period were identified from the electronic anesthesia records. Subsequently, the anesthesia methods were reviewed in the electronic anesthesia records, and cases where anesthesia was administered according to the protocol relevant to this study were extracted. Patients who underwent preoperative sedation, intubation, mechanical cardiopulmonary support, anesthesia method deviations, or the maintenance of anesthesia using methods outside the scope of this study were excluded. Patients whose medical records were incomplete were also excluded. All patients were eligible for the study, and their electronic medical records were analyzed. Propensity score matching in a 1:1 ratio was performed using the four variables of the PONV risk score (Apfel score) as covariates. They were categorized into the remimazolam group (R group) and the dexmedetomidine–propofol group (DP group) based on the anesthesia method used. Data were collected on demographics [age, gender, body mass index (BMI), smoking status, and history of motion sickness or PONV, New York Heart Association Functional Classification (NYHA)], comorbidities (hypertension, ischemic heart disease), anesthesia duration, surgery duration, intraoperative infusion volume, blood gas analysis, and postoperative status. The occurrence of nausea and vomiting, as well as the use of antiemetic medications, were obtained from medical records and drug prescription histories. The rates of PONV served as the primary outcome. The secondary outcomes were the nausea score and the dose of antiemetics (metoclopramide or granisetron) administered within 48 h of surgery.

### 2.1. The Definition of PONV

The terms were defined as follows: Vomiting is the forced expulsion of stomach contents from the mouth. Nausea is a subjective feeling of discomfort accompanied by an urge to vomit. Postoperative nausea and vomiting (PONV) occurs within 48 h of surgery [5].

### 2.2. The Definition of Nausea Score

The term “nausea” was defined in the “Common Terminology Criteria for Adverse Events v5.0”, published by the Cancer Therapy Evaluation Program of the National Cancer Institute [21]. Nausea is classified into four grades. Grade 0 shows the absence of nausea, while Grade 1 is described as “Loss of appetite without alteration in eating habits”, Grade 2 is classified as “Oral intake decreased without significant weight loss, dehydration or malnutrition”, and Grade 3 as “Inadequate oral caloric or fluid intake; tube feeding, total parenteral nutrition (TPN), or hospitalization indicated”. We defined these grades as nausea scores, with Grade 1 as Nausea Score 1, Grade 2 as Nausea Score 2, and Grade 3 as Nausea Score 3 for our evaluation.

### 2.3. The Extraction of PONV

The presence or absence of PONV and the number of vomiting events were extracted from nursing records documented in the electronic medical records. In the ICU, the severity of PONV was assessed and recorded by nurses at intervals of no less than every 6 h. Vomiting events were documented in real time by the nursing staff. In the general ward, the presence of nausea and the number of vomiting events were recorded by nurses at each shift as part of their routine observations. These assessments were standardized and incorporated into the clinical pathway, preventing omissions in documentation. The use of antiemetics was extracted from the prescription records in the electronic medical records. The nausea score was subsequently assessed for patients who exhibited PONV, with the assessment conducted by the researchers. To ensure objectivity in the evaluation, the presence of nausea and the amount of food intake, key indicators for determining the nausea score, were assessed by nurses. Food intake was evaluated by nurses on a scale from 1 to 10, reflecting the percentage of food consumed during each meal, and these values were recorded in the EMR for each meal. Whether total parenteral nutrition (TPN) or tube feeding was administered was verified based on the prescription records in the electronic medical records. A change in food intake compared to preoperative levels was categorized as Grade 2 if it had decreased, and Grade 1 if it remained unchanged. If inadequate oral intake necessitated the use of TPN or tube feeding, it was classified as Grade 3.

### 2.4. Anesthesia Methods

We followed our institution’s anesthetic protocol. Patients undergoing TAVR were not given premedication. Medications such as angiotensin-converting-enzyme inhibitors, angiotensin II receptor blockers, angiotensin receptor neprilysin inhibitors, and oral antidiabetic drugs were discontinued on the day of TAVR. Following the patient’s positioning on the operating table, we attached the electrocardiography, pulse oximetry, noninvasive blood pressure monitoring, and bispectral index (BIS; Aspect Medical Systems, Norwood, MA, USA). Before anesthesia induction, arterial blood gas analysis was performed with an arterial pressure line secured under local anesthesia. Anesthesia was maintained with either remimazolam and remifentanil or dexmedetomidine, propofol, and remifentanil. For remimazolam and remifentanil (R group), remimazolam was administered as a loading dose of 12 mg/kg/min and then maintained at 1 mg/kg/h after loss of consciousness. For the dexmedetomidine, propofol, and remifentanil (DP group), dexmedetomidine was given as a loading dose at 4 μg/kg/h for 10 min, followed by a maintenance dose of 0.7 μg/kg/h. Additionally, propofol was administered as a single 20 mg dose during the dexmedetomidine loading phase and as needed thereafter, as determined by specific procedural criteria and signs of arousal. Ultrasound-guided central venous catheterization was performed using combined local anesthesia following a loss of consciousness. Intraoperative monitoring was conducted using transthoracic echocardiography; there was no use of transesophageal echocardiography. Respiration was controlled through spontaneous breathing, and the upper airway was secured with a jaw elevation device (Hypnoz, West Brooklyn, IL, USA). Intraoperative analgesia was provided through the continuous infusion of 0.03 μg/kg/min remifentanil and intravenous acetaminophen (15 mg/kg) for postoperative pain management. Heparin (100 units/kg) was given after femoral artery cannulation to extend the activated clotting time. Dexamethasone sodium phosphate (6.6 mg) and granisetron (1 mg) were given intravenously at the start of surgery to prevent PONV. The bispectral index (BIS) monitor was used to keep the anesthetic depth within a specific range, and the respiratory rate during spontaneous breathing was kept between 10 and 25/min. Noradrenaline was started at a dose of 100 μg/h if the mean blood pressure fell below 65 mmHg during anesthesia. The norepinephrine preparation (Alfresa Phalma Co., Osaka, Japan) used was norepinephrine hydrochloride, which was prepared according to the Japanese Pharmacopeia. This preparation contained 1 mg/mL of norepinephrine. It was diluted 100-fold with normal saline for use. The anesthesia was discontinued after the TAVR valve was deployed. Flumazenil (0.5 mg) was given through the central venous line to the R group immediately after dressing. The patients were transferred to the intensive care unit after surgery and stayed overnight before being transferred to the general ward the next day.

In our facility, we made a transition from the dexmedetomidine–propofol method to the remimazolam method at a specific point (November 2020) during the study period. Thus, we did not select between the two anesthesia methods for any particular reason.

### 2.5. Statistical Analysis

The continuous variables were analyzed using the Wilcoxon signed-rank test. The Chi-square test was used for nominal scales, and Fisher’s exact test was applied to ordinal or nominal scales with expected frequencies of less than 5 to compare the R group and DP group. Propensity score matching was utilized to correct selection bias and confounding factors in constructing the Apfel score for predicting the PONV risk [16].

The factors considered were gender, history of motion sickness or PONV, nonsmoking status, and postoperative opioid administration. Because of the low surgical invasiveness of TAVR, intentional opioid administration for postoperative analgesia was not practiced in our institution. Since no patients received opioids postoperatively, the “intended postoperative opioid use” item on the Apfel score was excluded from the matching criteria. First, the covariates between the two groups were balanced using propensity score matching. All patients were matched in a 1:1 ratio based on their nearest neighbor. The caliper for nearest neighbor matching was 0.2. JMP for Mac (version 17.0.0, SAS Institute Inc., Cary, NC, USA) was used in the analysis. At the start of our study, determining a sample size proved difficult due to the lack of prior research comparing remimazolam and dexmedetomidine in TAVR sedation. To address this, we chose four of the Apfel score risk factors for PONV as covariates, excluding postoperative opioid use, which was not considered in this study. The minimum number of cases was then determined by multiplying the number of covariates by 15. Furthermore, our sample size was deemed adequate in comparison to the paper on sample sizes for studies utilizing propensity score matching [22]. To assess the robustness of the matching process, sensitivity analysis was performed using inverse probability weighting (IPW).

## 3. Results

**Figure 1:**
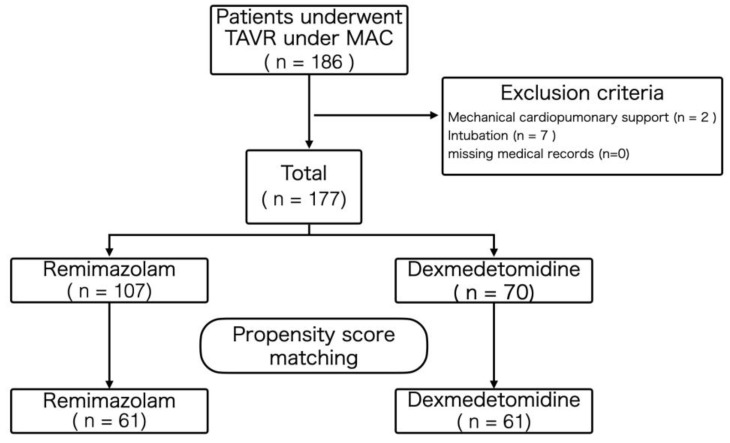
Flow chart of the study. TAVR: transcatheter aortic valve replacement, MAC: monitored anesthesia care.

A total of 186 TAVR patients were enrolled. Based on the exclusion criteria, nine cases were excluded because TAVR was performed under general anesthesia due to preoperative mechanical circulatory support (*n* = 2) or intubation (*n* = 7). There were no cases excluded due to anesthesia method deviations. A total of 177 cases were included in the analysis (Figure 1), with 107 (64.1%) patients undergoing the MAC with remimazolam protocol and 70 (35.9%) patients receiving the MAC with dexmedetomidine–propofol protocol. The patients were divided into two groups based on their anesthesia method: remimazolam (R) and dexmedetomidine–propofol (DP). Following propensity score matching, 61 patients were assigned to the R group and 61 patients to the DP group (Figure 1). This number of cases met the pre-determined minimum requirement of 60 cases per group. All patients underwent TAVR using a transfemoral approach. Table 1 depicts the patient characteristics used for propensity score matching. Table 2 shows the main patient characteristics after propensity score matching. The patients’ main characteristics before propensity score matching are also displayed in the Supplemental Table S1.

Table 3 contains additional information on patient backgrounds, intraoperative data, length of intensive care unit stay, and number of days from surgery to discharge. There were no significant differences in patient backgrounds between the two groups. There were no significant differences in anesthesia time (80 [71.5, 92.5] min vs. 86.5 [68.75, 106.25] min, *p* = 0.145), intraoperative fluid volume (1050.0 [800, 1255] mL vs. 1310 [887.5, 1900] mL, *p* = 0.165), and intraoperative remifentanil use (80 [60, 104] μg vs. 68.3 [44.2, 101.6] μg, *p* = 0.172).

The incidence of PONV within 48 h did not differ significantly between the two groups (R group: 3 out of 61 patients [4.92%] vs. DP group: 2 out of 61 patients [3.28%], *p* = 0.817). Furthermore, there was no significant difference between the two groups in terms of the occurrence of PONV within 48 h (*p* = 0.095) (Table 3). However, in the R group, two patients experienced multiple episodes of PONV. Fortunately, there was no missing data in these outcomes. However, in the R group, two people reported vomiting and nausea five or more times. In both groups, two patients took antiemetic medication within 48 h of surgery (Table 3). In the nausea scores, there was no significant difference between the two groups (*p* = 0.19) (Table 3). However, in the R group, there were two patients with a score of 1, one with a score of 2, and two in the DEX group with a score of 1 (Table 3). The length of intensive care unit stay and number of days from surgery to discharge had no significant differences between the two groups.

The incidence of PONV in the IPW analysis remained similar after propensity score matching (Table 3 and Supplementary Tables S2 and S3), as analyzed using EZR (Saitama Medical Center, Jichi Medical University, Saitama, Japan), a graphical user interface for R (The R Foundation for Statistical Computing, Vienna, Austria).

## 4. Discussion

We have introduced an original MAC protocol using a combination of remimazolam, flumazenil, and remifentanil for TAVR. In a previous report, we demonstrated that our protocol resulted in stable circulatory dynamics during anesthesia, and rapid, reliable awakening within approximately one minute after the administration of flumazenil, even in high-risk patients, classified as being physical status four by the American Society of Anesthesiologists (ASA), who underwent TAVR [3]. These findings reinforced previous reports [20,23,24,25] regarding the safety of remimazolam in high-risk patients, including those who are elderly or frail. Furthermore, rapid awakening facilitates prompt neurological assessments postoperatively, which may support the early detection of neurological complications [26,27] and improve prognosis [28] in the TAVR procedure, which has the risk of cerebral embolism. However, previous reports did not evaluate the complications associated with our protocol. Therefore, in this study, we focused on examining PONV, a typical adverse event associated with anesthesia.

In this study, we aimed to show that MAC with a combination of remimazolam, flumazenil, and remifentanil was not inferior to the dexmedetomidine-based conventional MAC in terms of PONV incidence. Our findings revealed a very low incidence of PONV (less than 5% in both groups), with no significant difference in incidence compared to the conventional method. Furthermore, there was no discernible difference in the severity of PONV. Therefore, there appeared to be no need to fear the risk of PONV in the use of MAC containing remimazolam, flumazenil, or remifentanil in TAVR.

Previous studies on the efficacy of remimazolam in treating PONV have yielded conflicting results. That is, while some papers [17,18,19] claim that remimazolam is superior or noninferior to other anesthetics in terms of PONV, others [15,16] argue that it is inferior. After reviewing the contents of these papers, it is clear that reports claiming the superiority of remimazolam over other anesthetics make limited use of flumazenil, whereas reports claiming its inferiority frequently include a higher proportion of antagonism with flumazenil. Midazolam, another benzodiazepine, has been reported on multiple occasions to potentially suppress PONV [29,30], and it is reasonable to expect that remimazolam, as a member of the same benzodiazepine class, would also act in this direction. Furthermore, several reports indicate that rapid antagonism by flumazenil may pose a risk for PONV [5,31]. Therefore, flumazenil’s rapid antagonistic effect may be a risk factor for PONV. However, the results of this study showed that even with the use of flumazenil, it did not significantly induce PONV. This study targeted an elderly population of TAVR patients, which distinguishes it from existing PONV research that has included younger cohorts. Analyzing the patient characteristics, it was evident that many TAVR patients had a lower risk of PONV. This fact may have influenced the outcomes of the study. Given its properties, remimazolam is believed to offer particular advantages for elderly patients, those with comorbidities, and individuals with significant frailty [3,23,24]. Consequently, the results of this study provide data on the patient population in which remimazolam exhibits its greatest efficacy, suggesting their relevance to actual clinical practice. There are still a lack of studies focusing on specific patient populations for remimazolam [20]. The results of this study demonstrate that, in high-risk patients, remimazolam can be used without fear of adverse PONV events, allowing for its benefits to be realized. Furthermore, these findings reinforce the evidence regarding the safety of both remimazolam and flumazenil, supporting their safe use in TAVR patients.

In the comparison of drug costs, the R group was more expensive than the DP group. This was due to the use of flumazenil and the dosage of remimazolam. In our protocol, for patients weighing around 50 kg, the additional cost was approximately 18% (around JPY 500, or about USD 3), while for patients with a larger body weight or longer surgery times, the cost increased further (approximately 99%, or JPY 2700, or about USD 18). This cost comparison is difficult to assess easily, as it is influenced by the healthcare system and drug pricing in each country. However, in the context of our country, the price difference was relatively small, less than USD 20. Therefore, considering the benefits, such as the early detection of neurological complications, we believe that the cost increase falls within an acceptable range.

This study has several limitations. First, it was designed as a retrospective analysis, which inherently limits the available data and introduces potential biases. Specifically, the extraction of PONV data was based on medical record documentation, which may not have captured all relevant instances or nuances of PONV. Additionally, eliminating selection bias regarding the choice between remimazolam and dexmedetomidine for anesthesiologists was challenging due to the retrospective nature of the design. However, during the study period, the anesthesia protocol was switched from the DP group to the R group on a specific day in November 2020. This allowed for a natural division of cases, where the first half of the study period involved the DP method and the second half used the R method, without the deliberate selection of the anesthesia method. As a result, this approach minimized selection bias regarding the choice of anesthesia.

Furthermore, this study focused on a population of very elderly patients undergoing TAVR, specifically high-risk, frail individuals with multiple comorbidities, to investigate the safety of remimazolam-based MAC in relation to PONV. While the results of this study may be applicable to elderly patients, caution should be exercised in generalizing these findings to broader populations, particularly younger individuals.

Additionally, the norepinephrine used in this study was in its hydrochloride form, and the dosage may not be directly comparable to that of other formulations of norepinephrine [32]. However, it is unlikely that this factor influenced the trends observed between the two groups.

Propensity score matching was employed to adjust for confounding variables; however, due to the lack of clear and consistent covariates, only four variables were matched, which may limit the effectiveness of this adjustment. Moreover, this was a small-scale, single-center study, and the results may not be directly applicable to other healthcare settings. This study was conducted at a high-volume sedated TAVR center, ensuring a high level of expertise among the surgical and anesthesia teams.

The sample size was not pre-determined due to the absence of prior studies comparing remimazolam and dexmedetomidine in this context. Consequently, the required sample size was estimated by multiplying the number of clear covariates included in the Apfel score (a risk prediction tool for PONV) by 15 cases. As a result, the study met the required sample size with 61 patients in each group. However, the small scale of this study suggests that further prospective research with larger sample sizes is needed to validate the findings and establish more robust conclusions.

In conclusion, in TAVR procedures, MAC with remimazolam had no difference in the incidence of PONV within 48 h compared to dexmedetomidine.

## Figures and Tables

**Table 1:** Patient characteristics before and after propensity score matching.

	Before Propensity Score Matching (*n* = 177)			After Propensity Score Matching (*n* = 61)		
	Remimazolam Group (*n* = 107)	DP Group (*n* = 70)	*p*-Value	Remimazolam Group (*n* = 61)	DP Group (*n* = 61)	
Gender						
Female	75 (70.1)	46 (65.7)	0.326	42 (68.8)	42 (68.8)	a
Nonsmoking status	63 (58.8)	18 (25.7)	<0.001	18 (29.5)	18 (29.5)	a
History of motion sickness or PONV	1 (0.9)	0 (0)	0.314	0 (0)	0 (0)	b
Postoperative opioids	0 (0)	0 (0)	N/A	0 (0)	0 (0)	b

Values are presented as *n* (%). The DP group is the dexmedetomidine–propofol group, PONV is postoperative nausea and vomiting. Propensity score matching was performed using the algorithm of nearest neighbor matching. Caliper width: 0.2. Sampling: no replacement. Composition ratio: one-to-one pair matching. Variables marked “a” were analyzed using the Chi-square test, and variables marked “b” were analyzed using Fisher’s exact test. N/A: Not Applicable.

**Table 2:** Patients’ characteristics after propensity score matching.

After Propensity Score Matching (*n* = 122)				
	Remimazolam Group (*n* = 61)	DP Group (*n* = 61)	*p*-Value	
Age (year)	85 (81.5, 89)	85 (81, 87)	0.222	a
Gender				
Female	42 (68.85)	42 (68.85)	1.000	b
BMI (kg/m^2^)	22.26 ± 3.52	22.72 ± 3.34	0.461	a
NYHA				c
1	0 (0)	2 (3.28)	0.320	
2	49 (80.33)	49 (80.33)		
3	10 (16.39)	7 (11.48)		
4	2 (3.28)	3 (4.92)		
History of hypertension	57 (93.44)	57 (93.44)	1.000	b
History of diabetes	8 (13.11)	18 (29.51)	0.025	b
History of ischemic heart disease	16 (26.23)	25 (40.98)	0.836	b
Euro 2 score (%)	4.248 ± 3.325	4.09 ± 5.509	0.854	a

Values are presented as *n* (%), median ± sd. BMI (body mass index), NYHA (New York Heart Association Classification), and Euro 2 score. Propensity score matching was performed using the nearest neighbor matching algorithm. Caliper width: 0.2. There were no replacements during sampling. The composition ratio is one-to-one pair matching. The DP group is the dexmedetomidine–propofol group. Variables marked as “a” were analyzed using the Wilcoxon signed-rank test, those marked as “b” with the Chi-square test, and those marked as “c” with Fisher’s exact test.

**Table 3:** Comparison of outcomes between the remimazolam and DP groups after propensity score matching and after IPW analysis.

	After Propensity Score Matching			After IPW for Sensitivity Analysis			
Variables	Remimazolam Group (*n* = 61)	DP Group (*n* = 61)	*p*-Value	Remimazolam Group (*n* = 111.4)	DP Group (*n* = 64.3)	*p*-Value	
Anesthesia time (minutes)	80.0 (71.5, 92.5)	86.5 (68.75, 106.25)	0.145	85.0 (73.0, 95.0)	91.0 (73.0, 114.0)	0.133	a
Infusion volume (mL)	1050 (800, 1255)	1310 (887.5, 1900)	0.165	1050 (800, 1250)	1350 (900, 1950)	0.016	a
Intraoperative remifentanil dose (μg)	80.0 (60.0, 104.0)	68.3 (44.2, 101.6)	0.172	80.0 (63.0, 110.0)	75.0 (45.0, 113.0)	0.297	a
Incidence of PONV within 48 h	3 (4.92)	2 (3.28)	0.817	2.4 (2.2)	2.7 (4.2)	0.944	b
The occurrence of PONV within 48 h			0.095			0.099	b
0	58 (95.08)	59 (96.72)		108.8 (97.8)	61.6 (95.8)		
1	1 (1.64)	2 (3.28)		1.8 (1.6)	1.9 (3.0)		
2	0 (0)	0 (0)		0.6 (0.6)	0.0 (0.0)		
3	0 (0)	0 (0)		0.0 (0.0)	0.8 (1.2)		
4	0 (0)	0 (0)		0.0 (0.0)	0.0 (0.0)		
5	1 (1.64)	0 (0)		0.0 (0.0)	0.0 (0.0)		
6	1 (1.64)	0 (0)		0.0 (0.0)	0.0 (0.0)		
Used antiemetic medication within 48 h postoperatively	2 (3.28)	2 (3.28)	0.690	2.79 (2.50)	2.71 (4.21)	0.094	a
nausea score			0.190			0.077	b
0	58 (95.08)	59 (96.72)		108.6 (97.8)	61.6 (95.8)		
1	2 (3.28)	2 (3.28)		2.79 (2.5)	2.71 (4.2)		
2	1 (1.64)	0 (0)		0.0 (0.0)	0.0 (0.0)		
3	0	0 (0)		0.0 (0.0)	0.0 (0.0)		
Length of intensive care unit stay (days)	2 (2, 2)	2 (2, 2)	0.139	2 (2, 2)	2 (2, 2)	0.234	a
Number of days from surgery to discharge	5 (4, 6.5)	5 (4, 6)	0.500	5 (4, 6)	5 (4, 6)	0.118	a

Data are presented as the median (IQR), *n* (%). The occurrence of PONV within 48 h and the nausea scores were analyzed using Fisher’s exact test. IPW is the inverse probability of treatment weighting, DEX is dexmedetomidine, and IQR stands for interquartile range. The DP group is the dexmedetomidine–propofol group. Variables marked as “a” were analyzed using the Wilcoxon signed-rank test and those marked as “b” with Fisher’s exact test.

## Data Availability

The datasets generated and/or analyzed during the current study are available from the corresponding author on reasonable request.

## Supplementary Materials

The following supporting information can be downloaded at: https://www.mdpi.com/article/10.3390/jcm14051759/s1, Table S1: Patients’ characteristics after propensity score matching; Table S2: Patient characteristics in the IPW analysis group; Table S3: The outcome measures in IPW analysis.

## Abbreviations

The following abbreviations are used in this manuscript:

PONV	Postoperative nausea and vomiting
MAC	Monitored anesthesia care
TAVR	Transcatheter aortic valve replacement
IRB	Institutional Review Board
BMI	Body mass index
NYHA	New York Heart Association Classification
BIS	Bispectral index
DEX	Dexmedetomidine
ASA	American Society of Anesthesiologists
