# Supplementary material for: Comparison of Postoperative Nausea and Vomiting Between Sedation with Remimazolam and Dexmedetomidine in Transcatheter Aortic Valve Replacement Patients: A Single-Center Retrospective Observational Study
Source: J Clin Med. 2025 Mar 5;14(5):1759. doi: 10.3390/jcm14051759 (PMC11900386; doi:10.3390/jcm14051759)
Supplement: Supplementary file 1 [file jcm-14-01759-s001.zip › jcm-3486609-supplementary.pdf]

Journal of Clinical Medicine

MDPI

Supplementary data

Table S1. Patient characteristics before and after propensity score matching.

	Before propensity score matching (n = 177)					After propensity score matching (n = 122)				
	n	Remimazolam group	n	D-P group	P-value	n	Remimazolam group	n	D-P group	
Gender	107		70			61		61		b
Female		75, 70.09		46, 65.71			42, 68.85		42, 68.85	
Male		32, 29.91		24, 34.29			19, 31.15		19, 31.15	
Age	107	84.32 ± 5.126	70	83.64 ± 5.41	0.39	61	84.85 ± 5.01	61	83.68 ± 5.45	a
BMI	107	22.03 ± 3.78	70	22.42 ± 3.61	0.49	61	22.26 ± 3.52	61	22.72 ± 3.34	a
NYHA	107		70		0.007	61		61		c
1		1, 0.93		3, 4.29			0		2, 3.28	
2		88, 82.24		56, 80.0			49, 80.33		49, 80.33	
3		12, 11.21		7, 10.0			10, 16.39		7, 11.48	
4		6, 5.61		4, 5.71			2, 3.28		3, 4.92	
The history of hypertension	107	97, 90.65	70	65, 92.86	0.78	61	57, 93.44	61	57, 93.44	b
The history of diabetes	107	16, 14.95	70	20, 28.57	0.99	61	8, 13.11	61	18, 29.51	b
The history of ischemic heart disease	107	25, 23.36	70	30, 42.86	0.99	61	16, 26.23	61	25, 40.98	b
Euro 2 score	107	4.53 ± 5.13	70	4.093 ± 5.32	0.58	61	4.248 ± 3.325	61	4.09 ± 5.509	a
The amount of remifentanil administrated during surgery	107	0.114 ± 0.11	70	0.082 ± 0.07	0.08	61	0.11 ± 0.127	61	0.085 ± 0.075	a

Values are presented as n (%), median ± sd. BMI (Body mass index), NYHA (New York Heart Association Classification), and Euro 2 score. Propensity score matching was performed using the nearest neighbor matching algorithm. Caliper width: 0.2. There will be no replacements during sampling. Composition ratio is one-to-one pair matching. D-P group is dexmedetomidine – propofol group. Variables marked as "a" were analyzed using the Wilcoxon signed-rank test, those marked as "b" with the chi-square test, and those marked as "c" with Fisher's exact test.

Table S2. Patient characteristics in the IPW analysis group.

Variables	P value	Standerdized Difference	
Used antiemetic medication within 48 hours postoperatively	0.555	0.094	c
Gender			b
Female	0.936	0.016	
Nonsmoking status	0.787	0.054	b
History of motion sickness or PONV	0.454	0.104	b

 https://doi.org/10.3390/jcm14051759

Age	0.402	0.133	a
BMI	0.666	0.105	a
NYHA	0.416	0.236	c
The history of hypertension	0.715	0.057	b
The history of diabetes	0.005	0.537	b
The history of ischemic heart disease	0.015	0.495	b
Euro 2 score	0.864	0.029	a
Anesthesia time (minutes)	0.133	0.458	a
Infusion volume (mL)	0.016	0.524	a
Intraoperative remifentanil dose (μg)	0.297	0.165	a
Length of Intensive care unit stay	0.113	0.234	a
Number of days from surgery to discharge	0.442	0.118	a

IPW is inverse probability of treatment weighting, PONV is postoperative nausea and vomiting, BMI is Body mass index, and NYHA is New York Heart Association Classification. Variables marked as "a" were analyzed using the Wilcoxon signed-rank test, those marked as "b" with the chi-square test, and those marked as "c" with Fisher's exact test.

Table S3. The outcome measures in IPW analysis.

Variables	n	Remimazolam group	n	D-P group	P-value	Standerdized Difference
The occurrence of PONV within 48 hours	111.4		64.3		0.907	0.099
0	108.8	97.8	61.6	95.8		
1	1.8	1.6	1.9	3.0		
2	0.6	0.6	0.0	0.0		
3	0.0	0.0	0.8	1.2		
4	0.0	0.0	0.0	0.0		
5	0.0	0.0	0.0	0.0		
6	0.0	0.0	0.0	0.0		
Nausea score	111.4		64.3		0.611	0.077
0	108.6	97.5	61.6	95.8		
1	2.8	2.5	2.7	4.2		
2	0.0	0.0	0.0	0.0		

Values are presented as n, %. IPW is inverse probability of treatment weighting, D-P group is dexmedetomidine – propofol group, and PONV is postoperative nausea and vomiting. P-value: Fisher's exact test.
